# Patient-Rated Performance of Home Health Agencies and Hospitalization Risk: A Propensity-Score Matched Analysis

**DOI:** 10.1093/geroni/igab046.2054

**Published:** 2021-12-17

**Authors:** Chenjuan Ma, Lisa Groom, Shih-Yin Lin, Daniel David, Abraham Brody

**Affiliations:** 1 NYU, NYU Rory Meyers College of Nursing, New York, United States; 2 NYU, New York, New York, United States; 3 New York University, New York, New York, United States; 4 NYU Hartford Institute for Geriatric Nursing, New York, New York, United States

## Abstract

Home health care is the most commonly used home- and community-based service to older adults “Aging in Place”. Patient experience of healthcare services is a critical aspect of patient-centered care. Indeed, policymakers have linked patient-rated quality of care to payment to healthcare providers. This study aimed to examine the association between patient-rated care performance of home health agencies and risk for hospitalization among Medicare beneficiaries. This study used several national datasets from 2016 and included 491,718 individuals from 8,459 home health agencies. Home health agencies’ performance was measured using patient experience star rating from the Home Health Consumer Assessment of Healthcare Providers and Systems (HHCAHPS). Propensity score matching was used to balance the differences in patient characteristics at baseline between those receiving care from high-performing home health agencies and those in lower-performing agencies. On average, patients were 80.5 years old, 65% female, 81% White, 10% Black, and 6% Hispanic, with 90% taking 5 or more medications. Patients had a mean score of 1.73 (SD=1.69) on the Charlson Index. Respectively, 10% and 16% of patients were hospitalized within 30 and 60 days of home health care initiation. Estimates of logistic regression after propensity score matching found that patients receiving care from lower-performing agencies were at similar risk for both 30-day (OR=0.99, p=0.817) and 60-day (OR=1.02, p=0.616) hospitalization following the start of home health care, compared to those in high-performing agencies. Our findings suggest discrepancies (or no relationship) between patient experience and objective outcomes of home health care.

